# Medical student lifestyle counselling for non-communicable disease: impact on students’ competence and patients’ health behaviors

**DOI:** 10.1186/s13584-022-00532-x

**Published:** 2022-05-24

**Authors:** Lilach Malatskey, Jumanah Essa-Hadad, Reut Eldar, Inna Filipov, Sophia Eilat-Tsanani, Mary C. J. Rudolf

**Affiliations:** 1 Department of Population Health, Faculty of Medicine, Bar-Ilan University Azrieli, Henrietta Szold 8, POB 1589, 1311502 Safed, Israel; 2 Clalit Health Services, Safed, Israel

**Keywords:** Lifestyle medicine, Lifestyle course, Medical students, Lifestyle patient counseling

## Abstract

**Background:**

Promoting healthy lifestyle is key to tackling lifestyle-induced diseases, yet many doctors feel unskilled and medical schools lack its inclusion in their curricula. The impact of a novel elective lifestyle course is described, where students provided 3 months’ coaching to at-risk patients.

**Methods:**

Students’ attitudes, competence and lifestyle were assessed pre- and post the 18-month course. Patients’ health measures and behaviors were measured. Student and patient views were ascertained.

**Results:**

Nineteen students, 13 controls, and 29 patients participated. Perception of physicians’ importance as lifestyle consultants increased in coaching students (mean ± SD 3.7 ± 0.4 vs. 3.2 ± 0.5; *p* = 0.05). Self-perceived competence remained high in coaching students (6.7 ± 1.8 vs. 6.7 ± 1.2; *p* = 0.66). Controls’ competence increased but did not attain coaching students’ levels (3.6 ± 2.1 vs. 5.5 ± 1.9; *p* = 0.009). Focus groups of students confirmed self-perceived acquisition of skills. More patients exercised (38% vs. 82.7%; *p* = 0.001); spent more time in physical activity (median mins/week + IQR) 25 + [0.180] vs. 120 + [45,300]; *p* = 0.039), and avoided less desirable foods, such as unhealthy snacks, sweets and drinks. LDL cholesterol showed declining trend. Patients highlighted students’ empathy and attentiveness; satisfaction was extremely high.

**Conclusions:**

The course successfully enhanced students’ counselling skills, with beneficial effects for patients. This model for teaching experience-based lifestyle medicine has potential policy implications in terms of promoting effective lifestyle counselling by future physicians.

## Background

According to the World Health Organization (WHO) two-thirds of diseases are the result of unhealthy lifestyle [1] and in 2014 the US Centers for Disease Control declared that seven of ten deaths are caused by chronic diseases [2]. Smoking, lack of physical activity, unhealthy diet, and alcohol lead to metabolic and physiological changes such as hypertension, obesity, hyperglycemia and cholesterol [1, 2]. An important approach is to focus on reducing risk factors such as tobacco, physical inactivity, unhealthy diet and alcohol [2]. One third of OECD countries have policies in place to promote healthy lifestyle in the primary care setting [3].

It is clear that lifestyle guidance by medical professionals is effective in promoting patient health [4] and patients tend to take their doctor’s views seriously and perceive their opinion as reliable [5]. Trust between physician and patient, good communication, the doctor's own lifestyle and views on promotion of health, all predict success in improving patients' health outcomes [5].

Despite the link between lifestyle and chronic disease, physicians often fail to provide guidance. For example, in an Israeli study where patients were questioned about guidance from their family doctor, only 12.5% of smokers were advised about smoking cessation and 43% with obesity about weight management [6]. Even less were given practical advice.

Several reasons have been given for this lack of guidance, and include time and faith in the effectiveness of counseling, low self-efficacy [7] and physicians’ own poor health behaviors, which themselves are a barrier to providing guidance [8].

To date, few medical schools focus on imparting lifestyle knowledge and skills to students. In 1985, the National Academy of Sciences recommended that 25-h should be dedicated to nutrition [9] yet by 2010, only 27% of US medical schools had incorporated nutrition courses into their curricula [10] and less than half addressed exercise prescription [11]. In 2017 the Bipartisan Policy Center called for inclusion of lifestyle medicine in undergraduate, graduate and continuing medical education [12], yet curricula today provide some limited theory and few include practical skills involving patients [13, 14].

The Bar Ilan Faculty of Medicine was established in 2011. Its curriculum incorporates some novel pedagogic approaches to lifestyle medicine, including a pre-clinical program with 28 h on lifestyle, health behaviors and small group sessions to practice counselling skills [15] but despite this, our students’ lifestyles during their first year deteriorate substantially [16].

In this paper we report another aspect of the curriculum—an elective experience-based course where pre-clinical students are linked to primary care patients with lifestyle-related conditions and provide practical guidance for behavior change. We evaluated how this form of experiential learning impacts students’ attitudes, self-efficacy and lifestyle, as well as patients' health behaviors.

## Methods

This mixed methods study was conducted between November 2015 and July 2017, involving students from three cohorts.

Students: Students in their first two years who opted to provide lifestyle guidance as part of a community placement course were recruited as participants. Students who chose another project in the course were recruited as controls. Their placements involved working with NGOs in a variety of fields such as the elderly, learning disabled, mental illness and adolescents at risk.

### Patients

Patients from Clalit Health Services, the largest insurer and provider in the city of Safed, were recruited by their family physician. Criteria included age 20–80 years, at least one chronic condition (such as hypertension, diabetes, BMI > 25, heart disease or dyslipidemia), who were deemed would benefit from lifestyle improvement. In addition, individuals coping with mental illness attending the Enosh Club, a local NGO, were approached. Patients received written information and signed a consent form.

### Training and support for students

Students underwent six 45-min training sessions on lifestyle guidance led by a medical lifestyle medicine specialist. The training involved theory and practice (see Box 1), and included case presentations, support and counseling. Students worked closely with the patients’ family doctor who gave guidance and approved goals. Students coached 2 to 3 patients over the 18-month course.

**Box 1:** Training and support for students: six sessions provided monthly

1.*Health behavior change*: Motivational interview fundamentals including the use of empathy, ways to evoke change, open questions and how to react to resistance. The trans-theoretical model of change: stage detection and relevant reaction and coaching tools—circle of health and SMART health prescription (subjective, measurable, attainable, realistic and time bound goals for health behavior change)
*Practical component*: Motivational interviewing exercise
2.*Socioeconomic, cultural, religious needs of the patient population*. The importance of cultural competence, and consideration of individuals’ cultural, religious, and social factors when providing counseling. Case studies were presented and students instructed on how to provide culturally appropriate guidance
*Practical component*: Meeting with clinic staff
3.*Health literacy*. The importance of ensuring that patients receive health education information that is clear and understandable. Students were given tools to use to help patients understand the counseling
*Practical component*: Practicing skills in a simulation session with actors
4.Healthy nutrition principles including counseling for patients with diabetes and hypertension
*Practical component*: Reading food labels exercise
5.*Approaches to obesity*, nutritional counseling for weight loss, healthy cooking
*Practical component*: using a food diary for documenting food intake and discussing change
6.*Summary and insights* – reflection on the experience, what was achieved, what went well, what needed improvement
*Practical component*: Focus group discussion

### Intervention (see Table 1)

**Table 1:** Coaching program delivered by students based on setting ‘SMART’ goals and practical work together

Session	Content
1. Introductory meeting and informed consent	An explanation of the project with student, doctor and researcher in the clinic Recent blood tests, medical condition reports and informed consent collected Questionnaire on patient's lifestyle and habits Patient given pedometer
2. Defining goals and objectives	A personal booklet was issued containing information, the health cycle and a table of change goals Patient and student agree on ‘SMART’ goals: e.g.: increase in X number of fruits and vegetables per day; decrease of X kg in weight per week; daily walk for X minutes etc
3–5. Working on the change process	Target set at each session At the following session, successes, challenges and potential solutions were discussed. Where necessary, assistance from the clinic dietitian and physician was sought Practical work on the goal; for example, joint supermarket shopping, food selection and reading food labels, healthy cooking or walks together
6. Conclusion	Completion of the follow up questionnaire Repeat blood tests Future plans for maintaining and promoting positive health behavior change

Students and patients met for one hour every two weeks on six occasions. Students guided patients towards a healthier lifestyle tailored to their medical condition. Students were randomly assigned, unless patients requested preference for gender or language competence (Hebrew/Arabic/Russian).

#### Outcome measures

##### Students

A questionnaire examining attitudes, self-efficacy and health behavior was administered pre- and post-course to participating students and controls. The questionnaire is a composite of the Israeli Ministry of Health National Survey of Knowledge, Attitude and Practices and the UK HENRY program [17, 18]. Questions included general health ranked on a Likert scale (1-usually not good, 5-excellent); stress (1-very little stressed, 5-very stressed); and behaviors including smoking, exercise and food consumption (0-never, 1-several times daily, 2-several times weekly, 3-several times monthly).

Attitudes toward physicians’ role were ascertained using 3 items from a validated instrument to assess physicians’ attitudes to lifestyle counseling [19] (ranked 1-strongly disagree, 4-strongly agree); students’ self-perceived competence in health behavior counselling was ranked 1-not at all, 10–very much. Focus groups were conducted to explore students’ experience, acquisition of skills and any impact on health behaviors.

##### Patients

Students administered a 27-item questionnaire to their patients. Questions relating to health behavior were as above. Blood pressure and BMI were extracted from the patient's medical file. Blood tests for glucose, HbA1c, lipid profile and liver function were requested pre- and one month post-completion of the coaching. Telephone interviews with patients were conducted by a researcher (RE) two weeks after completion of coaching. The semi-structured interview included patients’ experience of sessions and content, and six questions on the impact coaching had on their understanding of importance of lifestyle, changes made, students’ attentiveness and empathy, and general satisfaction (using a 5-point Likert scale (1-strongly disagree, 5-strongly agree)).

#### Data analysis

##### Quantitative

Continuous variables with a normal distribution were compared by t-test for independent samples; non-normally distributed ordinal variables by the Mann–Whitney test, and categorical variables using Chi-squared or Fisher's exact test. Wilcoxon test was used to compare pre- and post-intervention variables for the coaching group.

##### Qualitative

Phone interviews and focus groups were recorded and transcribed. Thematic framework analysis was conducted on the focus group transcripts to elicit views regarding experience-based learning, changes in perceived competence, impact on students’ health behaviors and views regarding the physician’s role in lifestyle change. Explanatory analysis was conducted on the telephone interviews, for perceptions of students’ abilities and attitudes, satisfaction with the program and attitudes to lifestyle improvement.

## Results

### Students

The characteristics of the 19 students and 13 controls are shown in Table 2. At baseline, controls had similar BMI and exercise levels, but more smoked (5vs1), had lower self-perceived health (3.1 + 0.4 vs 4 + 0.7), *p* = 0.02)) and felt more stressed (3.6 + 0.4 vs 2.8 + 0.9, *p* = 0.01).

**Table 2:** Comparison of students’ BMI, Sleep Behavior, Smoking, Health Status and Stress Levels at start and end of the course

	Intervention (n = 19)			Control (n = 13)			Intervention vs controls
	Pre	Post	*p*	Pre	Post	*p*	*p*
BMI (kg/m^2^)	22.3 (2.6)	22.6 (2.8(	0.34	22.4 (2.4)	22.6 (2.4)	0.27	0.56
# Hours of sleep at night	6.8 (0.6)	7.0 (0.6)	0.31	6.3 (0.8)	5.9 (0.9)	0.09	0.03
Perceived health status*	4 (0.7)	3.8 (0.8)	0.25	3.1 (4)	3.3 (3)	0.6	0.25
Perceived stress levels**	2.8 (0.9)	3.1 (0.6)	0.13	3.6 (4)	3.6 (3)	0.76	0.38
Physical activity (minutes/week)	142.6(125.7)	167.3 (126.5)	0.22	103 (82.9)	77.3 (64.7)	0.17	0.08

*Perceived health status: 1=very poor; 2=satisfactory; 3= good; 4= very good; 5= excellent

**Perceived stress levels: 1= very slightly stressed; 2= slightly stressed; 3= moderately stressed; 4= stressed; 5= extremely stressed

Over the subsequent 18 months controls slept on average 1 h less (*p* = 0.03) and coaching students were more than twice as active as controls, although this failed to reach statistical significance. No significant difference between groups was observed in BMI, self-perceived health or stress.

#### Attitudes towards the doctor's role

At baseline, both coaching and control students saw that their role as doctors would be to treat disease and provide lifestyle guidance (3.4 + 0.5; control 3.3 + 0.5); and thought that patients expected their doctor to set an example through their own health behaviors (3.5 + 0.5; control 3.2 + 0.7). Over the 18 months views diverged significantly (*p* < 0.05) regarding ranking the importance of the doctor’s role in lifestyle guidance. Coaching students increased their ranking (3.4 + 0.5 to 3.7 + 0.4; *p* < 0.05) while controls showed little change (3.3 + 0.6 to 3.2 + 0.5).

#### Competence in lifestyle guidance (see Fig. 1)

**Fig. 1:**
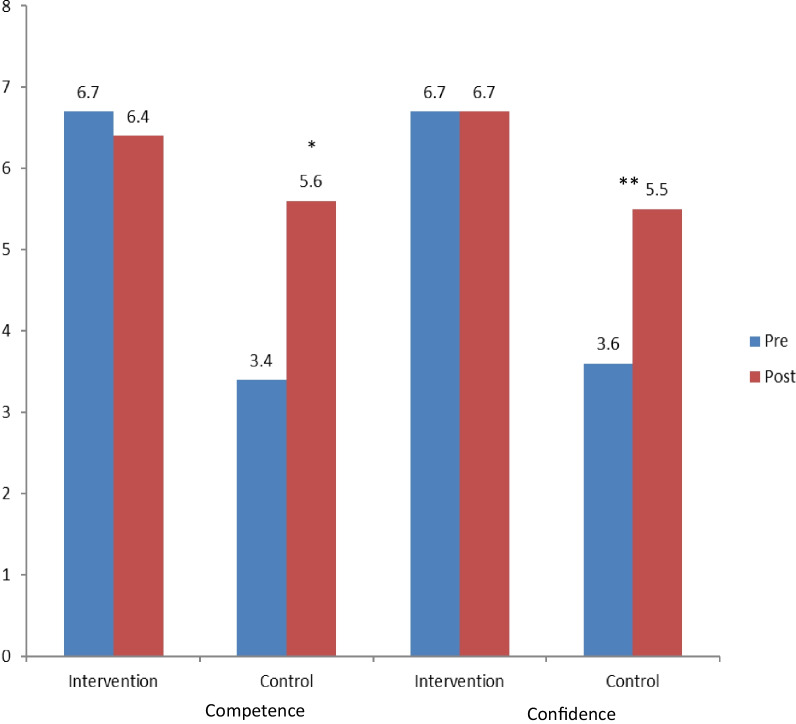
Self-reported competence and confidence in lifestyle guidance in coaching and control students at baseline and follow up at 18 months: At baseline coaching students’ competence and confidence were significantly greater than controls (*p* < 0.001, *p* = 0.009 respectively). Their scores showed no change over 18 months while control students' competence and confidence increased significantly (*p = 0.003 and **0.007 respectively)

At baseline, coaching students reported significantly greater perceived competence (6.7 + 1.4 vs 3.4 + 2; *p* < 0.001) and confidence (6.7 + 1.8 vs 3.6 + 2.1; *p* = 0.009) than controls. Over the 18 months the coaching group maintained their competence and confidence. The controls reported an increase in competence and confidence but still did not attain the coaching group’s levels (see Fig. 1).

#### Coaching students’ views

Themes from the three focus groups, in which all 19 students participated, included views on experience-based learning, competence/confidence, lack of knowledge and skills, change in attitudes, frustration at extent of lifestyle change achieved, and logistic challenges.

### Experience-based learning

The course provided students with their first opportunity to meet patients. They reflected on the benefits of experience-based learning over classroom learning, and its impact on understanding, awareness and the personal qualities required when caring for patients.

"It may also be that if I were not in the "lifestyle group" ….. I'm still not sure I would understand it, internalize it or remember ... now I guess I'll remember..." (female student 2016)

" it just gave me courage or attitude, it's not something I'd know how to do... It's also the first time I talked to a patient ... and to talk about these issues ... I would not have known how to do it..." (female student 2016)

### Confidence and competence

The students described how they had gained confidence and competence in the skills they needed. This included motivational approaches, coaching techniques, and different communication methods.

"It really opens up the thought processes, even if it's not someone who wants to hear, I at least have a positive attitude, I know how to deal with it... that's how it feels ...". (male student 2014)

"It's mainly the confidence to talk about it with people, that you know that you've already worked with a few patients... This is the most significant in my opinion…" (male student 2014)

Some acknowledged that despite their increase in knowledge it often remained inadequate for the complex task they had undertaken.

"I feel that I lack knowledge of certain subjects such as nutrition, smoking... I feel that I lack more tools... It's like, I acquired knowledge and gained access but am still missing things. " (male student 2015)

### Attitudes, understanding and the challenges of lifestyle change

Very clearly attitudes towards the doctor’s role and lifestyle changed through the experience. In general, their understanding about health behaviors increased substantially and they gained a greater appreciation of how challenging lifestyle change can be.

"For me at least it has changed… Now it is obvious to me that if someone came to me and tells me that he smokes, there is room to try and see if he is willing to make a change, and not to assume that that is how he is and that he really wants to stay this way …" (male student 2014).

“I saw how important it was to talk to the patient about his lifestyle. ….. As a doctor I do think that it's important to work and try as much as possible ... I don’t mean to nag in a way that will not help... But to try to do what you can... " (male student 2016)

Some students acknowledged their prior commitment to healthy living and felt that while they benefited from the course, their attitudes had not changed.

"If you ask if something has changed in my attitude, then no, I already believed that a doctor should be very pro-lifestyle in the first place." (male student 2015)

### Frustration

While much of the experience of lifestyle counselling was gratifying, students expressed frustration at how hard it was to help some patients find the motivation to change, compounded by expectations that greater changes were achievable.

‘…you ask him ‘have you perhaps thought of giving up smoking’. He tells you ‘What?!! It helps me!’ . You see his assertiveness…. So, you begin to doubt in his ability and willingness to change, and your own ability to help him. (male student 2015)

“all the approach that we bring: we want the person to be healthy and we want to help him… he does not, in truth, want help…. he is not interested in how to get there (male student 2015)

### Logistic challenges

Logistic challenges in fulfilling their commitments to patients included difficulties in finding the time to meet with patients on a regular basis in light of their burdensome studies. Disappointment when patients dropped out was tangible, although this rarely occurred beyond the initial session.

### Patients (see Table 3)

**Table 3:** Change in BMI and health behaviors in patients who were coached by medical students (n = 29)

	Before (N = 29)	After (N = 29)	*p*
BMI (mean (SD))	33.4 (5.5)	32.9 ( 5.0)	0.07
*Physical activity*			
Patients engaging in regular physical activity	11 (38%)	24 (82.7%)	< 0.001
Minutes physical activity/week (median + [IQR])	25 + [0,180]	120 + [45,300]	0.039
# of patients smoking	3	3	ns
*Biochemical parameters (mean (SD))*			
Fasting glucose (n = 22)	135.5 (40)	125.7 (44.4)	0.49
Total cholesterol (n = 24)	174.3 (25.5)	171.4 (39.6)	0.17
LDL cholesterol (n = 22)	96.2 (18.2)	91.8 (27.6)	0.05
HDL cholesterol (n = 22)	47.6 (6.6)	49.7 (14.5)	0.31

50 patients were recruited; 21 discontinued after the first session leaving 29 who completed six sessions. Mean age was 56.9 + 10.1 years, 15 were married, 19 were women. No significant sociodemographic or medical differences were found between patients who completed the coaching and those who dropped out.

At baseline mean BMI was 33.4 + 5.5, three were smokers, 11 had smoked previously, 15 had diabetes, 3 heart disease, 12 hypertension, 10 dyslipidemia and 5 a history of cancer. Targets chosen by patients included weight loss (n = 17), improved dietary habits (n = 11), more exercise (n = 8), diabetes control and symptoms (n = 11), alcohol reduction (n = 1).

### Impact of coaching on patients’ health and lifestyle (Table 3)

No significant change was found in BMI. There was a significant increase in physical activity, both in numbers engaged in physical activity and time devoted per week. There were also changes in reported dietary consumption with increased fruit (7 (24%)), salad (6 (21%)) and cooked vegetables (6 (21%)), decrease in savory snacks (10(34%)), sweets/chocolates (9 (31%)), sweet drinks (8 (28%)), fried foods (7 (24%)) and cookies/cakes (6 (21%)). There was a tendency towards improved biochemical measures although these did not reach statistical significance. No change occurred in stress, smoking, and reported general health.

The telephone survey following coaching showed very high satisfaction. Patients felt that the program improved their health and increased their knowledge about health issues. They highlighted how students’ empathy and attentiveness influenced their ability to make lifestyle changes: students’ attentiveness was ranked at 4.9 (+ 0.2); the extent to which coaching should be a regular service offered by the clinic at 4.7 (+ 0.7); and general program satisfaction at 4.6 (+ 1.0). 18 wanted to participate again and 17 responded that they would recommend the coaching should be offered routinely by the clinic.

## Discussion

This study examined the implementation of an innovative program in which pre-clinical students were introduced to patients with chronic diseases, received basic training in lifestyle medicine and guided patients towards improving their lifestyle as a way to treat their disease. They met with patients for six face-to-face sessions and experienced primary care with real patients, aiming to benefit the patients’ health. Positive findings were found for both students and patients.

Focus groups indicated that the experience-based course gave students the confidence to talk to patients about lifestyle, theoretical knowledge was internalized and useful tools acquired including the ability to conduct a motivational interview with reasonable confidence. Patients highlighted that students’ empathy and attentiveness influenced their ability to embark on a healthier lifestyle.

At the start, coaching students reported more confidence and competence in their ability to deliver lifestyle guidance. They also reported better health, feeling less stressed and smoked less. They, and the controls, had similar positive views regarding the doctor’s role in providing lifestyle guidance and patients’ expectations regarding doctors’ own health behaviors.

Over the 18 months coaching students’ attitudes diverged from controls regarding the importance of the physician as a lifestyle consultant. Coaching students also tended to improve their health behaviors, at least in terms of sleep and physical activity.

A key aim of the course was to increase students’ competence in lifestyle guidance. At first appearance, the coaching students’ failure to increase their self-efficacy was disappointing, particularly as control students' self-efficacy increased. However, this needs to be examined in the light of the ‘built-in gap’ between self-efficacy following theoretical learning and its actual application [20]. Students commonly encounter a fall in self-efficacy due to a lack of correspondence between their knowledge and the complexity of implementation [20]. This gap is often accompanied by stress, anxiety, shock and fear of harming patients [21]. The maintenance of a sense of self-efficacy in our students may therefore be considered an achievement, and perhaps their lack in rise of stress levels too. It is noteworthy that although controls reported increase in self-efficacy (whether illusionary or not) they did not achieve the coaching students’ levels.

Results from patients were also encouraging. They undertook more exercise and reported desirable changes in their dietary consumption. Despite the small sample size, biochemical parameters and BMI tended towards improvement too. Their satisfaction with the program and the students was very evident.

As educators we were gratified by the course’s success, however this was echoed less strongly by the students who described frustration at the slowness and size of change. No doubt expectations were naive alongside a lack of appreciation of how extremely challenging health behaviors can be. Clearly lifestyle training must involve realistic expectation for change.

Other examples of training preclinical students in lifestyle counselling [13, 22] have been reported, but on the whole are intended for educational purposes rather than service. They include two didactic sessions to develop prevention-oriented skills for health maintenance [23] practice on relatives/friends [13], diabetic patients [24] (three meetings) and geriatric patients [22] (one meeting). Patients' outcomes showed no change. Our course seems to be the only program that is consistent with a recent recommendation advocating for students to be given responsibility as health trainers to promote effective experiential learning [25]. It is gratifying that Bar Ilan University Azrieli Medical School now includes lifestyle guidance as a required component in its curriculum and all clinical students are assessed on their competence in counselling an appropriate in-patient at the end of their Internal Medicine rotation.

Physicians’ and medical students’ personal health behaviors serve as positive predictors of their views regarding counseling for lifestyle [8]. Deterioration in medical students’ lifestyle has been well documented by us [16] and others [24, 26] so the positive impact on students’ lifestyles is important for professional as well as personal development, especially in view of students’ study loads and levels of stress.

There are limitations to the study, the principal being the small sample size and lack of randomization. Indeed, there was evidence of selection bias, with the intervention students being highly motivated and more confident and competent regarding lifestyle guidance from the start. Recruitment of patients proved challenging and greater ‘through put’ would have enhanced the project, however the 60% completion rate is no mean achievement, especially as drop out almost always followed the introductory session. Pedometers were an integral part of the intervention, so some of the satisfying increase in lifestyle may not be attributable to the counselling alone. Some outcomes were based on self-report, although patients' blood tests were objective and despite small patient numbers almost reached statistical significance. Lastly, limited resources meant that patients could not be followed over time to ascertain sustainability of their improvements in health.

### Health policy implications

Our study demonstrates that the skills of practicing lifestyle medicine can be incorporated into medical studies. This is crucial given the current epidemiology of illness, with so many chronic conditions caused or exacerbated by poor lifestyle. We know from smoking cessation studies that even brief advice and warning by physicians can lead to significant and long-term change in smoking behaviors [27]. Lifestyle counseling must therefore be seen as an essential part of physician and other health professional interactions with patients and students must be provided with training and practical experience during their studies.

Experience based learning in lifestyle medicine is in line with the drive to transform medical education at all levels into becoming competency-based rather than knowledge-based [28]. Lifestyle medicine skills are already an explicit requirement; the General Medical Council in the UK, for example, demands that doctors in training must ‘demonstrate basic principles of public health, including promoting health and wellbeing, nutrition, exercise and illness prevention’[29].

Twenty years ago, the American Medical Association declared its support for legislation that incentivizes and provides funding for the inclusion of lifestyle medicine education in medical school education [30]. More recently, a symposium of leading health organizations, was convened by the Bipartisan Policy Center in the USA, calling for the inclusion of nutrition and physical activity at all levels of medical education [31]. Other action has included a demand for state and federal support for impactful and lasting change within the delivery of medical care, with the initiation of a think tank with the remit of opening communication, informing local- and national elected officials, and addressing potential necessary policy challenges [13]. Our study, although modest, is important. as we have shown that it is possible to train medical students in lifestyle medicine skills without allocation of significant resources. Indeed, it highlights the importance of making lifestyle counseling a required component of the medical education curriculum, and also has relevance to Israeli Ministry of Health guidelines regarding the employment of clinical students as physician assistants [32].

## Conclusion

The novel training program examined in this study preserved participating students’ high self-efficacy in accompanying patients in lifestyle change, their appreciation of the physician's role as a lifestyle consultant and had a positive effect on students’ and patients’ health behaviors. It demonstrated that it is feasible to impart knowledge and skills of lifestyle medicine combined with support and guidance during medical studies. Further study is required on the impact of the course now that it is a required component of the curriculum in our medical school.

The study has implications at a policy level in terms of lifestyle medicine training, and competency-based education. Engagement of physicians is an essential component of any multi-sectoral response to preventing and managing non-communicable diseases. To date this is often lacking, and the course provides a model for influencing future physicians’ attitudes and skills towards guiding patients to successfully introduce lifestyle change into their lives.

## Data Availability

Not applicable.

## Abbreviations

NGO: Non-governmental organization
BMI: Body mass index
